# Heatwave-Induced Thermal Stratification Shaping Microbial-Algal Communities Under Different Climate Scenarios as Revealed by Long-Read Sequencing and Imaging Flow Cytometry

**DOI:** 10.3390/toxins17080370

**Published:** 2025-07-27

**Authors:** Ayagoz Meirkhanova, Adina Zhumakhanova, Polina Len, Christian Schoenbach, Eti Ester Levi, Erik Jeppesen, Thomas A. Davidson, Natasha S. Barteneva

**Affiliations:** 1 Department of Biology, School of Sciences and Humanities, Nazarbayev University, 010000 Astana, Kazakhstan; adina.zhumakhanova@nu.edu.kz (A.Z.); polina.len@alumni.nu.edu.kz (P.L.); 2 Department of Ecoscience, Aarhus University & Center for Water Technology (WATEC), 8000 Aarhus, Denmark; eel@ecos.au.dk (E.E.L.); ej@bios.au.dk (E.J.); thd@bios.au.dk (T.A.D.); 3 Sino-Danish Centre for Education and Research, Beijing 100049, China; 4 Department of Biological Sciences and Centre for Ecosystem Research and Implementation, Middle East Technical University, 33731 Erdemli-Mersin, Turkey; 5 Institute of Marine Sciences, Middle East Technical University, 33731 Erdemli-Mersin, Turkey; 6 Institute for Ecological Research and Pollution Control of Plateau Lakes, School of Ecology and Environmental Science, Yunnan University, Kunming 650500, China; 7 The Environmental Research and Efficiency Cluster, Nazarbayev University, 010000 Astana, Kazakhstan

**Keywords:** thermal stratification, *Microcystis*, algicidal bacteria, long-read sequencing, microbial communities, imaging flow cytometry, FlowCam

## Abstract

The effect of periodical heatwaves and related thermal stratification in freshwater aquatic ecosystems has been a hot research issue. A large dataset of samples was generated from samples exposed to temporary thermal stratification in mesocosms mimicking shallow eutrophic freshwater lakes. Temperature regimes were based on IPCC climate warming scenarios, enabling simulation of future warming conditions. Surface oxygen levels reached 19.37 mg/L, while bottom layers dropped to 0.07 mg/L during stratification. Analysis by FlowCAM revealed dominance of Cyanobacteria under ambient conditions (up to 99.2%), while Cryptophyta (up to 98.9%) and Chlorophyta (up to 99.9%) were predominant in the A2 and A2+50% climate scenarios, respectively. We identified temperature changes and shifts in nutrient concentrations, particularly phosphate, as critical factors in microbial community composition. Furthermore, five distinct *Microcystis* morphospecies identified by FlowCAM-based analysis were associated with different microbial clusters. The combined use of imaging flow cytometry, which differentiates phytoplankton based on morphological parameters, and nanopore long-read sequencing analysis has shed light into the dynamics of microbial communities associated with different *Microcystis* morphospecies. In our observations, a peak of algicidal bacteria abundance often coincides with or is followed by a decline in the Cyanobacteria. These findings highlight the importance of species-level classification in the analysis of complex ecosystem interactions and the dynamics of algal blooms in freshwater bodies in response to anthropogenic effects and climate change.

## 1. Introduction

Cyanobacterial harmful algal blooms (CyanoHABs) deteriorate the water quality of freshwater bodies by depleting dissolved oxygen [1], increasing water turbidity [2], and producing a variety of toxic metabolites [3,4]. The frequency of these blooms is rapidly increasing in many regions of the world, driven by rising anthropogenic activities and climate change [5,6]. Climate change projections from the Intergovernmental Panel on Climate Change (IPCC) are commonly used to model future environmental conditions. The high-emission scenario (A2) predicts a global mean temperature increase of 3 °C by the end of 2100 [7]. In addition to the projected rise in global air temperature, climate models also predict an increase in the frequency, duration, and intensity of extreme heat events such as heatwaves. Global heatwaves have intensified [8,9,10,11], further altering CyanoHAB dynamics leading to the global dominance of *Microcystis* spp. [12,13].

Heatwaves significantly affect aquatic ecosystems increasing surface water temperature and shifting oxygen dynamics, increasing greenhouse gas emissions, and leading to euthrophication and higher levels of cyanobacterial biomass. With changing temperatures, the lake stratification patterns may change more frequently and with longer duration in shallow lakes [8,9]. The role of heterotrophic bacteria associated with Cyanobacteria and phytoplankton can vary greatly depending on environmental factors such as heatwaves, euthrophication, and nutrient levels [6,14].

Microbial interactions, particularly between phytoplankton and heterotrophic bacteria, are crucial to the dynamics of aquatic ecosystems. Cyanobacteria shape their surrounding microenvironment by producing extracellular polysaccharides [15], limiting light penetration and oxygen concentration [7], and altering levels of CO_2_ and pH [16]. The organic matter released from phytoplankton after algal death impacts bacterial communities in a species–specific manner [17]. In turn, heterotrophic bacteria can affect bloom development and stability by producing growth factors like vitamin B12 [18,19,20,21,22,23] and siderophores that bind iron utilized by microalgae, thus facilitating trace metal availability [24,25]. They also regenerate nutrients from organic materials [26,27,28,29], supporting the long-term survival of phytoplankton members. These mutualistic and antagonistic interactions are often highly specific to particular species or strains. However, much remains unknown about how these relationships shift in response to stressors like heatwaves or nutrient enrichment. Changes in the composition of the phytoplankton community have been shown to correlate with changes in bacterial community composition [30,31]. In freshwater systems that serve as sources of drinking water, it is essential to understand how bacteria control harmful algal bloom (HAB) [32,33].

The marked increase in year-to-year temperature variations and the frequency of heatwaves pose a serious threat to lake ecosystems [34,35,36] and draws attention to the need to understand algal–microbial interactions during extreme climate events and their further impact on lake ecosystems. Mesocosm experiments offer a valuable opportunity to model algal–microbial interactions in larger volumes and under conditions that are closer to the natural environment, compared to lab cultures [37,38,39,40]. Currently, short-read sequencing of hypervariable regions of the bacterial *16S rRNA* gene is the most commonly used to characterize bacterial community composition [41,42,43,44,45,46]. However, short-read sequencing technologies, such as those based on Illumina instruments, are limited to targeted regions of the *16S rRNA* gene at hundred base pairs (bp) length of reading. Commonly used PCR primer sets leave “blind’ gaps and may affect microbial diversity results [47]. While short-read sequencing of the *16S rRNA* gene is widely used, it is limited by short-read lengths. In contrast, long-read sequencing platforms such as Oxford Nanopore (UK) or PacBio (USA) allow for full-length 16S sequencing, as read lengths about 1500 bp are sufficient to cover the entire 16S rRNA gene. This capability may ultimately provide species-level resolution [48,49,50]. Additionally, imaging flow cytometry (IFC) enables the high-throughput morphological classification of phytoplankton based on cell and colony structure. Despite these potential benefits, there are few studies that integrate next-generation sequencing with flow cytometry and phenotyping for the co-occurrence analysis of microbial communities [51]. Moreover, there is a notable lack of research utilizing IFC to investigate host–microbe associations in aquatic systems.

In this study, we combined long-read nanopore sequencing and IFC to investigate bacterial communities associated with freshwater phytoplankton under contrasting climate change scenarios. Special attention was given to *Microcystis*, a morphologically diverse and ecologically significant cyanobacterium. Although *Microcystis* is commonly classified into morphospecies (e.g., *M. aeruginosa*, *M. novacekii*), recent genomic studies have demonstrated high genetic similarity (>99% 16S identity) between the morphospecies/morphotypes [13,52]. Nevertheless, morphospecies remain useful as operational units for ecological monitoring due to their distinct morphology and colony structure. In this work, we refer to *Microcystis* morphospecies as visually distinguishable morphological forms recognized via IFC, acknowledging that these may not accurately reflect true phylogenetic divergence [52].

We hypothesize that species–specific interactions with dominant phytoplankton groups influence bacterial community structures, and that changes in the physico-chemical environment, such as temperature and nutrients, impact these relationships over time. In particular, this study aims to investigate significant shifts in the structure of bacterial communities associated with potentially toxic *Microcystis* spp., *Cryptomonas* spp., and members of the Chlorophyta phylum, using a mesocosm that simulates the thermal stratification caused by short heatwaves.

## 2. Results

### 2.1. Microbial and Phytoplankton Community Composition Is Influenced by Temperature

**Figure 1:**
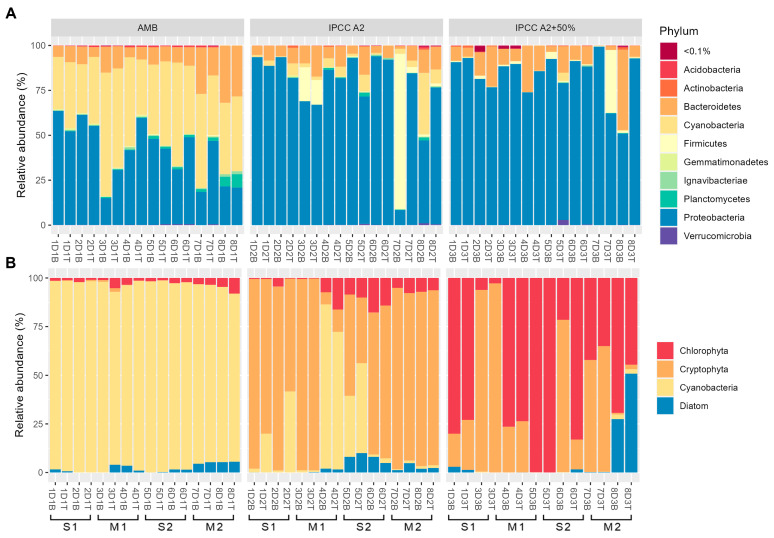
Relative abundances of dominant bacterial phyla detected using full-length 16S rRNA sequencing (**A**) and phytoplankton groups detected using FlowCAM-based IFC (**B**) across three temperature regimes (AMB—ambient, IPCC A2—high emission scenario, IPCC A2+50%—climate scenario version 50% with 50% higher temperature increase). S1 and S2—first (weeks 1 and 2) and second (weeks 5 and 6) stratification periods, respectively, and M1 and M2—first (weeks 3 and 4) and second (weeks 7 and 8) mixing periods, respectively. Labels “T” and “B” on the *x*-axis represent surface and bottom samples, respectively.

We assessed the composition of microbial and phytoplankton communities using a combined approach of full-length *16S rRNA* sequencing and imaging flow cytometry—mNGS-IFC (microbial NGS combined with imaging flow cytometry). Temporal changes in temperature and oxygen levels are outlined in Suppl. Figure S1. Overall, we observed a decrease in temperature across all mesocosm tanks throughout the eight-week experiment. Notable differences in the temperature of the surface and bottom layers were observed during the first stratification period. Similarly, oxygen levels were generally higher in the surface water compared to the bottom layers during both stratification periods, indicating varying degrees of stratification in the tanks. In tank D1 (ambient temperature regime, AMB), the temperature remained consistent between the surface and bottom layers, with minor stratification observed during weeks 1 and 2. However, more pronounced vertical stratification was observed in oxygen levels, which ranged from 11.41 to 19.37 mg/L at the surface and from 0.07 to 14.88 mg/L in the bottom layers. Both 16S sequencing and IFC-based analysis revealed the dominance of Cyanobacteria (ranging from 27.2% up to 68.8% based on 16S rRNA and from 85.7% up to 99.2% based on IFC) throughout the experiment in the phycosphere of tank D1 (Figure 1). The most abundant cyanobacterial species identified via sequencing was *Microcystis aeruginosa*, among 52 detected cyanobacterial species. The IFC analysis further distinguished five *Microcystis* morphospecies: *M. novacekii*, *M. ichtyoblabe*, *M. smithii*, *M. aeruginosa*, and *M. wesenbergii* as reported earlier (Suppl. Figure S2) [53,54]. Following Cyanobacteria, Proteobacteria comprised the second largest bacterial phylum in this tank (14.9% up to 63.5%) and a total of 618 identified species. Apart from cyanobacterial and proteobacterial phyla, Bacteroidetes ranked as the third largest phylum in this tank, with relative abundance ranging from 5.4% to 31.9%, and 222 identified species.

Similarly, tank D2 (IPCC A2 scenario, moderate warming [7]) exhibited temperature-based stratification during the first stratification period (weeks 1 and 2). Notable differences in oxygen levels between the layers were observed during both stratification periods. The temperatures recorded ranged from 18.8 °C to 33.1 °C. Proteobacteria were the dominant group, with their relative abundance fluctuating from 8% to 93.8%. This was followed by Bacteroidetes, which accounted for 1.4% to 17.2% of the microbial community (Figure 1). Cyanobacterial presence was variable and generally lower compared to D1. 16S-based data indicated a cyanobacterial peak during week 8 (the end of the second mixing period)—34.2%, with Nostocaceae being the dominant family. In contrast, IFC recorded a peak during week 4, coinciding with a slight temperature increase (Suppl. Figure S1). Unlike tank D1, *Aphanocapsa* sp. was found to be the major contributor to cyanobacterial abundance. Lastly, the analysis of major phytoplankton groups revealed a large proportion of the Cryptophyta phylum with varying dominance throughout the experiment. *Cryptomonas* sp. was identified as the dominant genus within this group. The peak abundance of Cryptophyta occurred at the beginning of the first mixing period (week 3) in both layers, with 98.8% (6793 particles/mL) in the surface layer and 98.9% (6839 particles/mL) in the bottom layer. Conversely, the minimum abundance (6%) corresponded to the increase in Cyanobacteria during week 4.

Tank D3 (under IPCC A2+5% scenario with high warming [7]) also exhibited strong stratification. The Proteobacteria phylum was the largest bacterial group in tank D3 (62.2–99.4%), while the presence of Cyanobacteria was minimal throughout all weeks as shown in Figure 1. The phytoplankton communities were predominantly composed of Chlorophyta, particularly *Micractinium* sp., which peaked during week 5 (99.9% in both surface and bottom layers), according to IFC analysis. Additionally, Cryptophyta was identified as the second largest phylum, peaking at 97.1% during week 3. Overall, the presence of Cyanobacteria in this tank was minimal.

**Figure 2:**
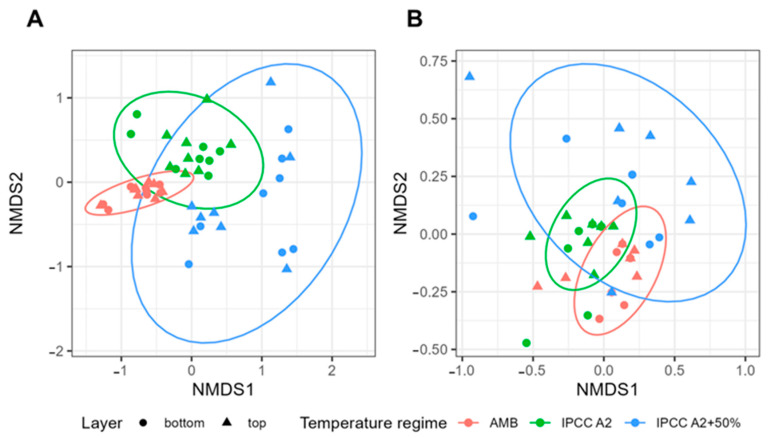
Non-metric multidimensional scaling (NMDS) analysis of community compositions throughout the experiment. NMDS ordination plots with stress values of 0.16 (**A**) and 0.14 (**B**) indicate the clustering of microbial (**A**) and phytoplankton (**B**) communities across temperature regimes. Each ellipse and color represent a different treatment group: AMB—red, IPCC A2—green, and IPCC A2+50%—blue. Ranked dissimilarities between all identified clusters were significantly different for both microbial and phytoplankton compositions (R = 0.54, *p*-value = 0.001 and R = 0.34, *p*-value = 0.001, respectively).

To investigate how treatment influences bacterial communities’ shifts, we applied non-metric multidimensional scaling (NMDS). The NMDS analysis further confirmed the differences in community compositions across the tanks (Figure 2). Distinct bacterial and phytoplankton communities developed at varying temperatures (AMB, IPCC A2, and IPCC A2+50% [7]) over the course of the experiment. Pairwise analysis of similarities (ANOSIM) revealed a significant difference between these groups (*p*-value < 0.05), demonstrating that the thermal regime was a major factor influencing community structure.

### 2.2. Network Analysis of Microbial Communities Across Varying Temperature Regimes

To explore how temperature affects microbial interactions, we constructed co-occurrence networks based on significant correlations (Spearman’s ρ ≥ 0.7, *p* < 0.05) for each mesocosm treatment. Network analysis provides insights into the potential ecological associations within microbial communities under different thermal regimes.

**Figure 3:**
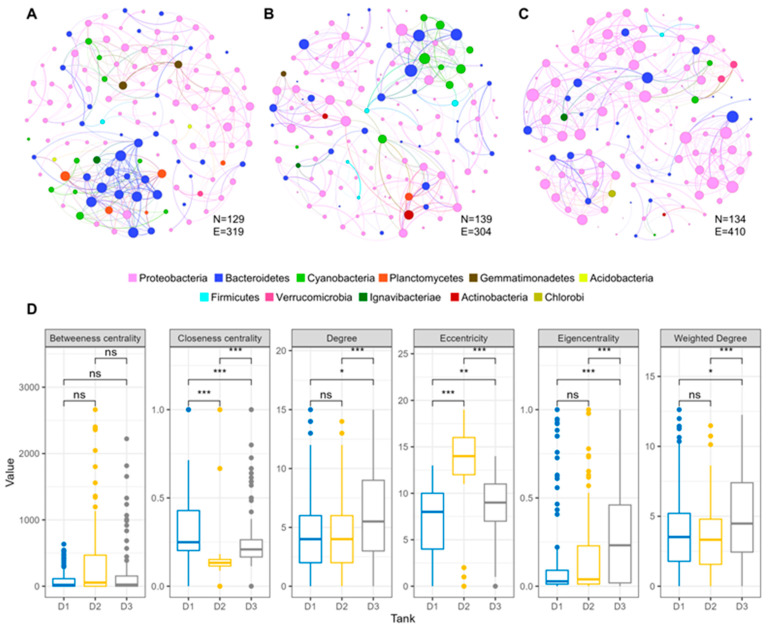
Network analysis of microbial communities across three temperature regimes based on correlation analysis (N = nodes; E = edges; *** = *p*-value < 0.001; ** = *p*-value < 0.01; * = *p*-value < 0.05; ns = not significant). Networks were constructed for communities at (**A**) AMB, (**B**) IPCC A2, and (**C**) IPCC A2+50% regimes. Network edges are weighted and represent a strong correlation (Spearman’s ρ ≥ 0.7, *p*-value < 0.05); the size of each node is proportional to the degree. (**D**) Differences in topological features of each constructed network at node level (represented by dots); pairwise comparisons based on Wilcoxon test.

The network created for the ambient temperature regime (AMB, tank D1) consisted of 129 nodes and 319 edges, with Proteobacteria (58.9%), Bacteroidetes (17.9%), and Cyanobacteria (7.7%) as the dominant phyla (Suppl. Table S1). In the moderate warming regime (IPCC A2, tank D2), we observed a slight increase in the number of nodes (139), but a decrease in edges (304), suggesting a reduction in strong co-occurrences. In contrast, the high warming regime (IPCC A2+50%, tank D3) had 134 nodes and 410 edges, indicating the highest average network degree (6.1) and suggesting a denser network structure and greater potential connectivity among microbial taxa (Figure 3A–C).

Further analysis revealed significant differences in several topological features at the node level of the obtained networks (Figure 3D). The IPCC A2+50% network had significantly higher values for node degree, weighted degree, and eigen-centrality compared to the other treatments. These metrics reflect the relative influence or connectivity of individual taxa within the community. The increased connectivity observed in this treatment suggests more complex or redundant interactions, potentially indicating that the microbial community is adapting to higher thermal stress. In contrast, the values of closeness centrality for the IPCC A2 network were significantly lower than those of the other networks, indicating lower-degree connections. Furthermore, significant differences in closeness centrality and eccentricity were identified between the networks, while no significant differences in betweenness centrality were found. Overall, the differences at the network level indicate shifts in community organization in response to thermal stress. Notably, Cyanobacteria played a relatively minor role in these networks under increased warming conditions, supported by the observed decline in their abundance.

### 2.3. Temporary Stratification Impacts Microbial Community Composition

Samples were collected from two depths to investigate the influence of temporary thermal stratification on microbial community composition. Communities were compared between stratified (weeks 1, 2, 5, and 6) and mixed (weeks 3, 4, 7, and 8) conditions within each mesocosm.

**Figure 4:**
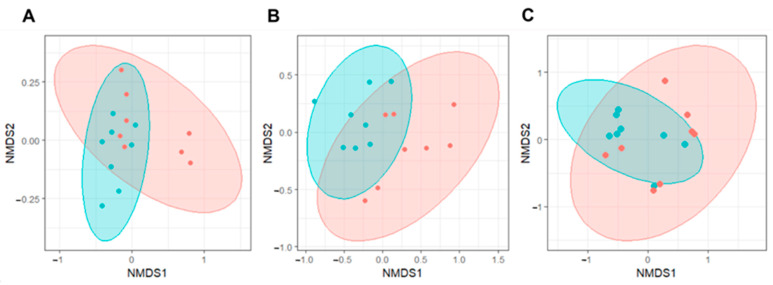
NMDS analysis of microbial community compositions detected using full-length 16S rRNA sequencing throughout the experiment. NMDS ordination plots indicate clustering of microbial communities across mixing conditions: mixing vs. stratification period across temperature regimes ((**A**)—AMB (R = 0.2185, *p*-value = 0.02), (**B**)—IPCC A2 (R = 0.3697, *p*-value = 0.004), (**C**)—IPCC A2+50%). Each ellipse represents a different treatment group: mixing—red, stratification—blue.

The overall composition in the mesocosm tanks during mixing was different from communities during stratification periods (Figure 4). NMDS analysis revealed a significant shift in community composition between stratified and mixed periods, particularly in tanks D1 (AMB) and D2 (IPCC A2) (Figure 4A,B). No significant difference was found for tank D3 (IPCC A2+50%).

**Figure 5:**
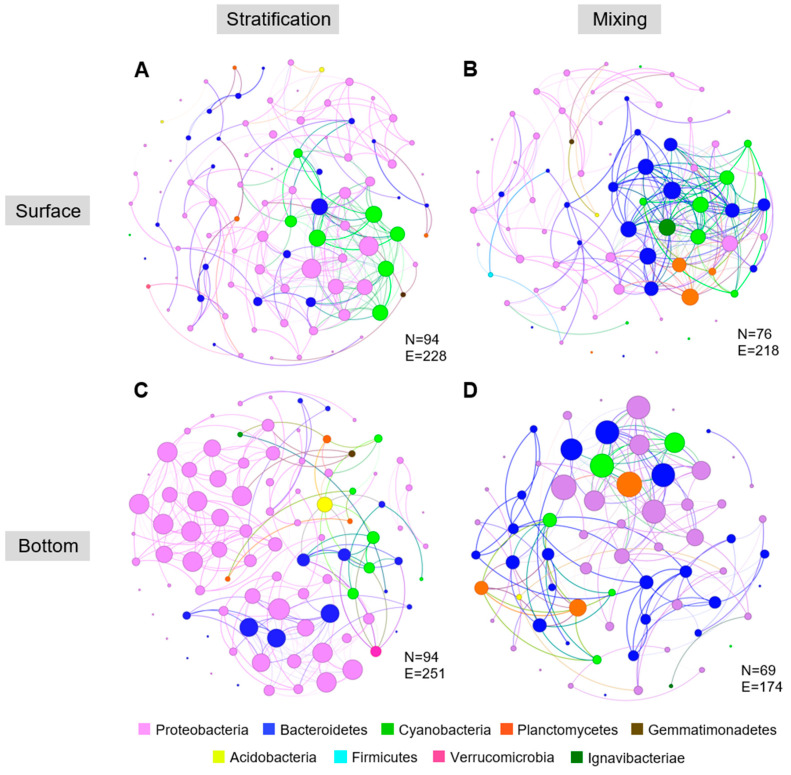
Network analysis of microbial communities in the tank with an ambient temperature regime (tank D1) across sampling layers and contrasting mixing conditions (N = nodes; E = edges). (**A**) Co-occurrence network of microbial communities at the surface layers during stratification and (**B**) mixing periods; (**C**) co-occurrence network of microbial communities at the bottom layers during stratification and (**D**) mixing periods.

A further analysis of co-occurrence networks in tank D1 revealed similar patterns (Figure 5). The constructed networks comprised 94 nodes and 228 edges for surface layers during stratification, 76 nodes and 218 edges during mixing, 94 nodes and 251 edges for bottom layers during stratification, and 69 nodes and 174 edges during mixing periods. The structure and connectivity of networks differed between stratified and mixed conditions. The average degree on the surface layer during stratification was lower (4.851) compared to mixing (5.737), indicating fewer interactions under vertical stratification in the tank. On the contrary, in the bottom layer, the average degree during mixing was slightly lower (5.043) than during stratification periods (5.34).

Taxonomic shifts were also observed during the stratified and mixed periods. The relative abundance of microbial communities at the class level (Suppl. Figure S3) revealed that the abundance of Alphaproteobacteria and Chitinophagia was significantly higher during stratification, while Ignavibacteria and Sphingobacteriia were more prevalent during the mixing period.

### 2.4. Environmental Drivers and Phytoplankton-Associated Microbial Communities

#### 2.4.1. CCA of Environmental Gradients

**Figure 6:**
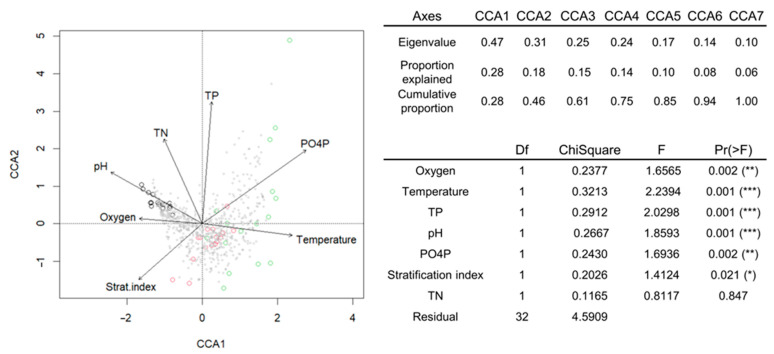
Canonical correspondence analysis (CCA) of microbial community composition and selected environmental parameters. Black circles correspond to samples from different dates with an AMB regime, red to the IPCC A2 regime, and green circles to the IPCC A2+50% regime. Statistical significance is indicated as follows: * *p* < 0.05, ** *p* < 0.01, *** *p* < 0.001.

Canonical correspondence analysis (CCA) was conducted to identify the environmental variables that influence microbial community structure. The factors included in the analysis were pH, oxygen levels, temperature, stratification index, total phosphorus (TP), PO_4_P, and total nitrogen (TN) levels (Figure 6). The first two constrained axes accounted for 26.8% of the total variance. The temperature and shifts in nutrient concentrations, particularly phosphate, were identified as critical factors in microbial community composition, particularly under the IPCC A2+50% regime.

#### 2.4.2. Species-Level Patterns in Dominant Bacterial Genera

**Figure 7:**
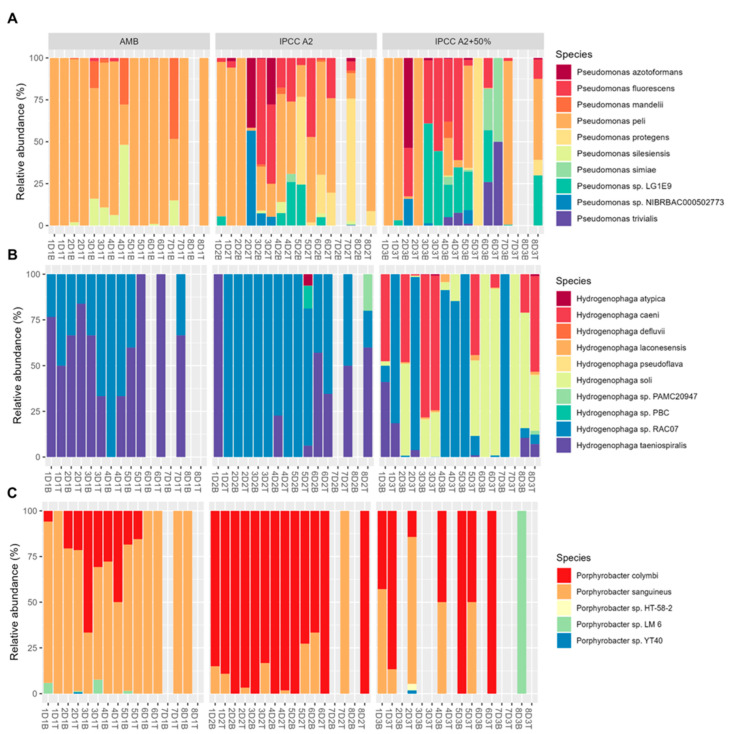
Species-level distribution of 3 common bacterial genera found in tanks D1, D2, and D3. (**A**) Distribution of members of Pseudomonas genera found in tanks D. (**B**) Distribution of members of *Hydrogenophaga* genera. (**C**) Distribution of members of *Porphyrobacter* genera. Temporal changes in species-level distribution are plotted on the *x*-axis, and relative abundance is plotted on the *y*-axis.

Among the identified bacterial genera, three were of particular interest: *Pseudomonas*, *Hydrogenophaga*, and *Porphyrobacter*. Although these genera were found in all mesocosm tanks, their species-level composition varied (Figure 7A). The species-level composition of *Pseudomonas* genera varied between the tanks. Indicator species analysis (ISA) revealed that *P. protegens* were strongly associated with tank D1, whereas *P. trivialis* and *P.* sp. LG1E9 were indicator species in tank D3. The species-level composition of *Hydrogenophaga* and *Porphyrobacter* genera also showed variability between the treatments (Figure 7B,C). *H. soli* were associated with tank D3, while *Hydrogenophaga* sp. RAC07 and *H. taeniospiralis* were prevalent in tanks D1 and D2, respectively. Among *Porphyrobacter* species, *P. sanguineus* and *P. colymbi* were found to be the indicator species for tanks D1 and D2, respectively. These results highlight the significance of full-length *16S rRNA* sequencing in detecting fine-scale shifts in bacterial communities and suggest that certain bacterial species may serve as bioindicators for specific environmental conditions.

#### 2.4.3. Cryptophyta-Associated Microbial Clusters

**Figure 8:**
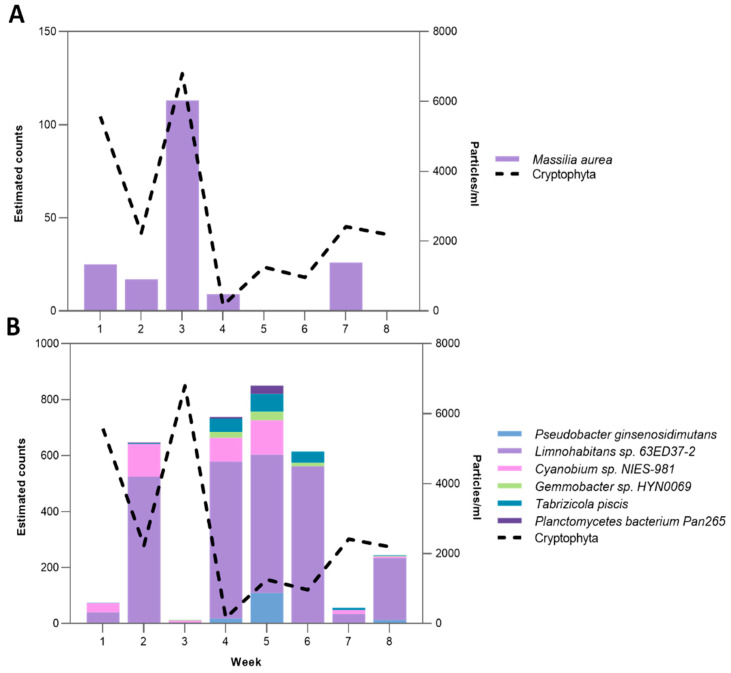
Dynamics of the Cryptophyta phylum and associated bacterial species. (**A**) *Cryptomonas* sp. absolute abundance against positively correlating (*p*-value < 0.05) *Massilia aurea* in surface layers of tank D2. (**B**) *Cryptomonas* sp. absolute abundance against negatively correlating (*p*-value < 0.05) microbial cluster in surface layers of tank D2. A negative correlation is shown by a red dashed line.

Given the dominance of Cryptophyta in tank D2, we examined associated microbial clusters. The data obtained indicated a significant positive correlation in the surface layer between the Cryptophyta phylum algae and *Massilia aurea* (r = 0.82, *p*-value < 0.05), a member of the Betaproteobacteria class. Common peaks between the two groups were found during weeks 1, 3, and 7 (Figure 8). In contrast, a negatively correlation with Cryptophyta microbial cluster (r = −0.78, *p*-value < 0.05) was identified during the weeks 2 and 4–6. This cluster was composed of members of the Betaproteobacteria, Alphaproteobacteria, and Chitinophagia classes, with *Limnohabitans* sp.63ED37-2 being the most abundant species (Figure 8B). The inverse relationship suggests possible antagonistic interactions, potentially driven by changes in the available nutrients.

#### 2.4.4. Chlorophyta-Associated Microbial Clusters

**Figure 9:**
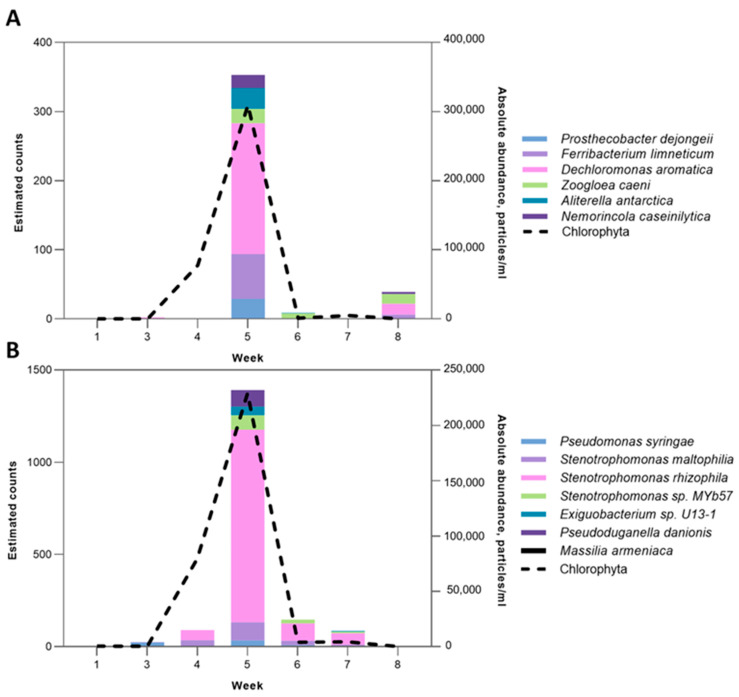
The dynamics of the Chlorophyta phylum and associated bacterial species. (**A**) Chlorophyta absolute abundance against the positively correlating (*p*-value < 0.05) microbial cluster in the surface layers of tank D3. (**B**) Chlorophyta absolute abundance against the positively correlating (*p*-value < 0.05) microbial cluster in the bottom layers of tank D3.

Tank D3 was dominated by members of the Chlorophyta phylum *Pediastrum* sp., *Scenedesmus* sp., and *Micractinium* sp., with *Micractinium* sp. being the most abundant genus. The relative abundance of Chlorophyta in tank D3 ranged from 2.7% to 99.9% in the surface water layer and from 6.2% to 99.9% in the bottom layer, peaking at week 5 (Figure 9). To construct microbial co-occurrence networks and further identify any significantly correlating microbial clusters against the Chlorophyta phylum, the Pearson correlation coefficient was calculated. The co-occurrence network analysis revealed strong positive correlations (r = 0.95–0.96, *p* < 0.05) between Chlorophyta abundance and distinct bacterial clusters in both surface and bottom layers (Figure 9). The cluster was composed of members of the Betaproteobacteria class, namely *Ferribacterium limneticum*, *Dechloromonas aromatica*, and *Zoogloea caeni*, along with microbes of the Verrucomicrobiia and Chitinophagia classes. Additionaly, significant positive correlations were identified in the bottom layers of tank D3 between Chlorophyta and one of the microbial clusters (r = 0.95). This cluster was composed of four members of the Gammaproteobacteria class (*Pseudomonas syringae*, *Stenotrophomonas maltophilia*, *Stenotrophomonas rhizophila*, and *Stenotrophomonas* sp. MYb57), the Betaproteobacteria class (*Pseudoduganella danionis* and *Massilia armeniaca*), and the Bacilli class (*Exiguobacterium* sp. U13-1).

### 2.5. Microcystis-Associated Microbial Clusters

**Figure 10:**
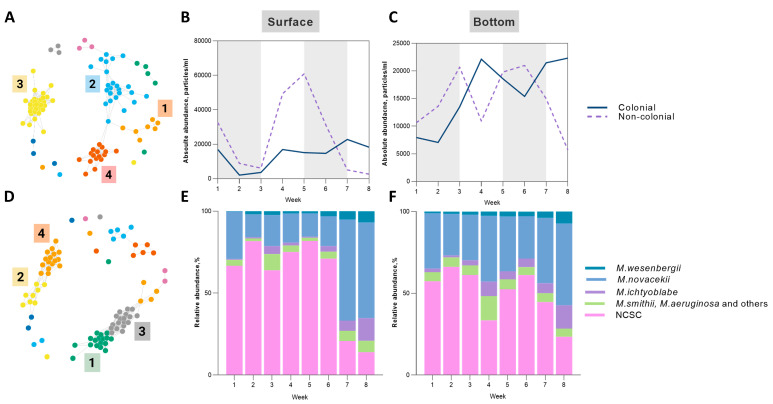
*Microcystis*-associated microbial clusters. (**A**,**D**) Co-occurrence network based on Pearson correlation coefficient (R > 0.75, *p*-value < 0.05) for surface samples—(**A**) and bottom samples (**D**) of tank D1, four major microbial clusters (1-4) were identified in each layer and color-coded separately for each cluster; (**B**,**C**) absolute abundance of colonial and non-colonial (NCSC) forms of *Microcystis* throughout the experiment in surface and bottom layers, shaded areas correspond to stratification periods; (**E**,**F**) relative abundances of *Microcystis* morphospecies and non-colonial forms throughout the experiment in surface (**E**) and bottom (**F**) layers.

Using IFC, we identified five morphologically distinct *Microcystis* morphospecies—*M. novacekii*, *M. ichtyoblabe*, *M. smithii*, and *M.aeruginosa*, *M. aeruginosa*, *M. wesenbergii* (Suppl. Figure S2) [53,54]. The classified *Microcystis* species were then divided into two main groups: colonial (consisting of five morphospecies) and non-colonial (NCSC) *Microcystis*. Figure 10B,C illustrate the changes in the absolute abundance of these two groups in the surface and bottom layers of tank D1. A peak of NCSC was observed in the surface layers (60,847 particles/mL) at the beginning of the second stratification period (week 5). However, during the start of the second mixing period (week 7), there was a significant decline in NCSC *Microcystis* in the surface layer (dropping to 5110 particles/mL), which was accompanied by a decrease in abundance in the bottom layer as well (from 14,959 particles/mL during week 7 to 5685 particles/mL by the end of the experiment). Overall, by the end of the experiment, the abundance of non-colonial *Microcystis* decreased, while the number of colonial forms increased.

The dynamics of separate morphospecies are illustrated in Figure 10E,F. During the experiment, varying levels of dominance were observed for *M. novacekii,* which was the most abundant morphospecies during the experiment in both layers. The peak abundance of *M. novacekii* occured during week 7, at the start of the second mixing period, with counts of 15,209 particles/mL in the surface level and 13,411 particles/mL in the bottom layer. *M. wesenbergii* and *M. ichtyoblabe* contributed almost equally to the total abundance of *Microcystis*.

A correlation analysis based on the Pearson correlation coefficient was conducted to determine microbial species that co-occurred throughout the experiment. Reads were first filtered to eliminate species with <1% relative abundance; obtained correlation coefficients less than 0.75 were filtered out (*p*-value < 0.05) prior to network construction. Resultant networks in tank D1 (Figure 10A,B) revealed eight microbial clusters with the largest number of members—four clusters for the surface and four for the bottom layers. The composition of each cluster is listed in Suppl. Tables S2 and S3.

**Figure 11:**
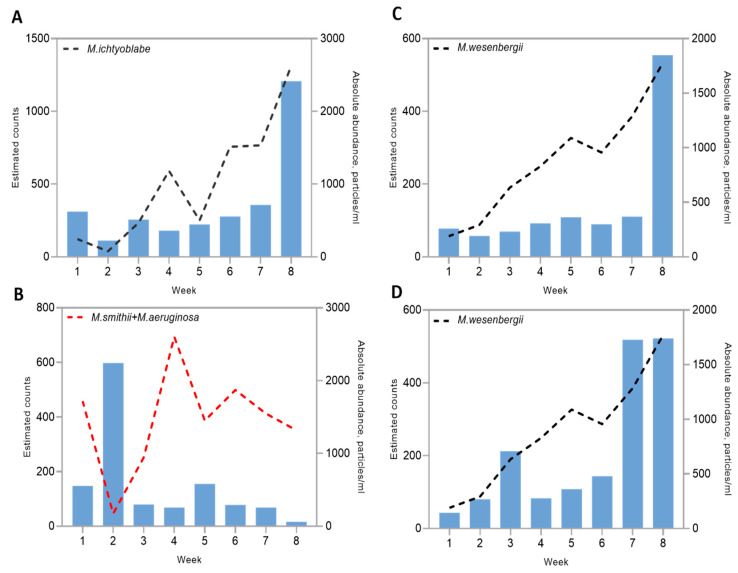
Dynamics of *Microcystis* spp. abundance against significantly (*p*-value < 0.05) correlated microbial clusters in tank D1. Blue bars represent the abundance of each significantly correlated microbial cluster. (**A**) *M. ichtyoblabe* absolute abundance against positively correlating microbial cluster 3 in the surface layers; (**B**) *M. smithii* and *M. aeruginosa* group absolute abundances against negatively correlating microbial cluster 4; (**C**) *M. wesenbergii* absolute abundance against positively correlating microbial cluster 1 in the bottom layers; (**D**) *M. wesenbergii* absolute abundance against positively correlating microbial cluster 3 in the bottom layers. A negative correlation is shown with a red dashed line.

The Pearson correlation coefficient matrix revealed significant correlations (*p*-value < 0.05) between some microbial clusters identified in the network analysis and *Microcystis* morphospecies. A strong positive correlation was identified between microbial cluster 3 and *M. ichtyoblabe* abundance, with a Pearson coefficient of r = 0.81. The peak abundance of *M. ichtyoblabe* was recorded during week 8, coinciding with the peak in the abundance of cluster 3 (Figure 11A). Cluster 3 included various members of the Bacteroidetes, Cyanobacteria, and Proteobacteria phyla (Suppl. Table S2). Notably, Sphingobacteriales were the largest order within this cluster, with 12 species identified. In addition to this, a significant negative correlation (r = −0.71) was found between cluster 4 and the cumulative abundances of *M. smithii* and *M. aeruginosa* morphospecies in the surface layers of tank D1 (Figure 11B). Cluster 4 mainly encompassed members of the Alphaproteobacteria class, specifically from the Rhodospirillales order (Suppl. Table S2). The main peak of this cluster corresponded with a decrease in the absolute abundance of *M. smithii* and *M. aeruginosa* morphospecies during the end of the first stratification period (week 2). On the other hand, the maximum abundance of *Microcystis* morphospecies during week 4 was accompanied by a decline in the abundance of the microbial cluster 4.

Positive correlations (*p*-value < 0.05) between the abundance of *M. wesenbergii* and identified microbial clusters were also found in the bottom layers of tank D1. In particular, positive correlations with microbial clusters 1 and 3 were identified (r = 0.76 and 0.81, respectively) (Figure 11C,D). Interestingly, the members of clusters 1 and 3 of the bottom layer (Suppl. Table S3) were similar to the members of cluster 3 in the surface layer, which had a significant positive correlation with *M. ichtyoblabe* abundance. Peaks in the abundances of clusters 1 and 3 coincided with a peak in the abundance of *M. wesenbergii* during week 8. Cluster 1 was composed of members of Proteobacteria, Planctomycetes, Cyanobacteria, and Bacteroidetes phyla. In contrast, cluster 3 was dominated by Bacteroidetes, with minor contributions from Proteobacteria, Planctomycetes, and Cyanobacteria. Differences between the clusters were observed during weeks 3 and 7, which marked the beginning of mixing periods, during which the abundance of cluster 3 increased. No significant correlations between the abundance of *M.novacekii* and the identified microbial clusters were found.

## 3. Discussion

The microalgae–microbiome interactions are central to natural aquatic ecosystems and artificial cultures [55,56]. The studies on phytoplankton–bacterial assemblages dominated by *Microcystis* spp. concluded that bacterial communities associated with *Microcystis* colonies are distinct from those associated with other Cyanobacteria and free-living communities [57,58,59,60,61]. However, few studies have focused on elaborating a detailed characterization of algal-associated microbes, and mostly in laboratory conditions [62,63,64], rarely by isolating bacteria during *Microcystis* bloom events [65]. Most existing studies addressed the interactions of microbiomes with microalgae at the class and genus levels [66].

The spatiotemporal dynamics of bacterial communities and individual bacterial ecotypes are essential to understanding bacterial–algal community composition and interactions. There are “generalist” bacterial species, which occur throughout the whole season, and “specialists,” which appear in significant numbers only for a limited amount of time or irregularly [67,68]. Heterotrophic bacteria in freshwater ecosystems are responsible for most organic matter cycling and a significant part of system respiration [69]. The co-occurring heterotrophic bacteria could have either negative or positive impacts on algal blooms, contributing to nutrient cycling, phytoplankton, and the lysis and degradation of toxins [70]. Field and laboratory studies revealed bacterial clusters attached to cyanobacterial and microalgal aggregates, creating an algal microenvironment or phycosphere [71] and synchronization between planktonic bacteria growth with phytoplankton bloom [72,73]. There is an increasing appreciation of short-term, ‘pulse’ heatwave events and their role in climate change. However, analysis of changes in the composition and biodiversity of lakes’ microalgal–bacterial communities’ is practically absent.

Sequence analysis of *rRNA* genes [74,75] and comparative analysis of marker genes [76] have revealed a distinct set of “freshwater-specific” bacterial taxa with consistent temporal differences in the composition complexity of bacterial communities’ [77]. Furthermore, most microbial community studies focus on either bacterial or eukaryotic communities, but investigations aimed at obtaining an integrated view of the temporal dynamics of changes, eventually revealing that the underlying ecological inferences are scarce [78,79], in particular for freshwater ecosystems [80,81]. An emerging approach to microbial community research based on NGS is “correlation networks” that can be used to determine drivers in environmental ecology and help researchers in hypothesis generation [78,82,83]. Recently, single *Microcystis* colonies’ microbiomes and *Microcystis*-epibiont communities were analyzed [84,85,86]. It was found that *Microcystis* blooms are accompanied by a diverse community of heterotrophic bacteria that play an important role in cyanobacterial bloom development and duration [85,86]. However, the molecular techniques used to characterize the *Microcystis*-associated microbiomes need more resolution in identifying the member bacteria at the species level [84]. Full-length sequencing of the 16S amplicons has been shown to facilitate microbiome characterization by reaching a deeper level of taxonomic resolution [87], and accuracy has been shown to be adequate for microbial diversity studies [88]. In the present study, we applied long-read nanopore-based NGS analysis of 16S amplicons and visualization-based IFC to simultaneously characterize the dynamics of diversity and co-occurrence in two domains of life, bacteria and Eukarya, in mesocosms with different regimes of temperature and mixing.

### 3.1. Microcystis-Associated Microbiomes

*Microcystis* is characterized by great phenotypic plasticity, and >50 *Microcystis* species have been identified by microscopy, often being referred to as morphospecies, including *M. aeruginosa*, *M. flos-aquae*, *M. ichthyoblabe*, *M. wesenbergi*, *M. novacekii*, and others [89,90]. However, a comparison of 16S rRNA species has revealed >99% similarity and inconsistency of physiological and genetic analysis, suggesting that some morphospecies represent a single species [13,91]. Lately, whole genome sequencing and phylogenetic clustering intended to differentiate *Microcystis* genospecies indicate significant future changes in *Microcystis* taxonomic classification [52,90], suggesting that different *Microcystis* morphospecies may be different morphotypes representing just one genetically consistent species, and their phenotypic plasticity is caused by environmental factors. These discrepancies between morphological and molecular biological identification were also confirmed by Schweitzer-Natan and co-authors, who found that *Microcystis* population in Lake Kinneret, where the composition and biomass of *Microcystis* have been recorded weekly for 50 years by microscopic identification. Thus, the dominant genotype in the years 2004–2016 was *M. aeruginosa,* but the most common species in 2016 was *M. flos-aqua*, comprising over 95% of the *Microcystis* biomass [92]. In our morphological analysis of *Microcystis* populations in LMWE mesocosm tanks, we observed periodical changes in *Microcystis* morphoforms detected by imaging flow cytometry [54], suggesting the existence of more variants of morphologically distinct *Microcystis* that are changing colonial structure in response to environmental parameters. However, research regarding the dynamics of microbiomes associated with different *Microcystis* morphospecies during algal blooms is practically absent.

The mNGS-IFC approach and network co-occuring analysis allowed us to identify and follow four clusters of heterotrophic bacteria associated with different *Microcystis* morphospecies. This permitted a correlation of the peaks of certain *Microcystis* morphospecies at different levels of the water column with the abundances of associated microbial clusters. *Microcystis* in the natural water environment tend to migrate vertically, regulating the buoyancy and changing cell density. We found that the vertically stratified distribution of Cyanobacteria affected the composition and dynamics of associated microbial clusters and the heterotrophic bacteria in these clusters. Moreover, the species composition of associated microbiome clusters differed between colonial and non-colonial forms.

Accumulating evidence suggests that there may be widespread metabolic interactions between *Microcystis* and associated microbiomes [14]. The initial phase of the bloom development of *Microcystis* coincided with an increase in microbial cluster numbers and included members of the ammonia-oxidizing Nitrosomonadales order (Cluster 1, *Methyloversatilis discipulorum*). Moreover, the decrease in the absolute abundance of *M. smithii* and *M. aeruginosa* morphospecies during the end of the first stratification period has coincided with an increase in the members of the hydrocarbon-degrading gamma-proteobacteria Xanthomonadales (Cluster 4; *Stenotrophomonas rhizophilia*) which is similar to the observation by Gutierrez and co-authors [93] regarding the structure of *Microcystis* bloom associated microbiomes.

We hypothesized that a high abundance of algicidal bacteria would coincide in time with *Microcystis* abundances decrease, and our findings confirmed these expectations. To date, several algicidal bacteria have been identified as being associated with cyanobacterial blooms, and these have been isolated and investigated [32,62,94,95]. The literature reports a high diversity of anticyanobacterial microbes, encompassing more than 50 genera. The majority of these belong to the genera Pseudomonas, Aeromonas, Acinetobacter, Citrobacter, among others, all of which are classified under different classes of Proteobacteria. Anticyanobacterial Actinomycetes include *Rhodococcus* sp., *Arthrobacter* sp., *Microbacterium* sp., and *Streptomyces* sp. Additionally, many Bacteriodetes such as *Pedobacter* sp. *Aquimarina* sp., Firmicutes including the Bacillus group, *Exiguobacterium* sp., and *Staphylococcus* sp., have shown high efficiency in inhibiting *Microcystis* growth [64]. In our studies, we observed a significant diversity among various *Pedobacter* spp. from Sphingobacteriales order associated with the collapse of *M. wesenbergii* bloom (*Pedobacter cryoconitis*, *Pedobacter* sp. PACM 27299, *Pedobacter mongoliensis*). The seasonal dynamics of *Microcystis* morphospecies and their microbial antagonists, along with the collapse of cyanobacterial blooms correlated with the growth of cyanolytic bacteria, have been described in several studies [96]. Our analysis yielded highly congruent results with early observations indicating that peaks in algicidal bacteria abundance coincide with or follow a decline in cyanobacterial blooms [97,98].

### 3.2. Cryptophyta- and Chlorophyta-Associated Microbiomes

The composition of Chlorophyta- and Cryptophyta-associated microbiomes was less complex than *Microcystis*-associated microbial clusters. The bloom development of Chlorophyta and Cryptophyta coincided with an increase in associated heterotrophic bacteria. The network inference analysis revealed that Massilia, a member of Oxalobacteriaceae, also had prevalently positive interactions with Chlorophyta, *Massilia armeniaca*, thus being associated with Chlorophyta at the bottom of the vertical water column. Our results are in accordance with previous findings of positive interactions between *Massilia* and Chlorophyta [99].

Early researchers described the microbiomes associated with *Cryptomonas* spp. [100,101]. Notably, Betaproteobacteria are abundant in many freshwater habitats and, in laboratory studies, often associate with algae such as *Cryptomonas* spp. [100,101]. In freshwater ecosystems, one of the key bacterioplankton groups within Betaproteobacteria is the genus *Limnohabitans*. Published studies on co-culture have demonstrated significant increases in the abundance of *Limnohabitans* strains when cultured alongside *Cryptomonas* sp. but not in cyanobacterial cultures (*Aphanizomenon* sp., *Dolichospermum* sp.) [101]. In our own research, we found that the microbial cluster associated with *Cryptomonas* sp. included *Limnohabitans* sp. (63ED37-2). Moreover, we noted that the peak abundance of the freshwater betaproteobacterium *Massilia aurea* coincided with a peak in *Cryptomonas* sp. abundance in tank D2. This finding supports early observations of Salcher and co-authors [102] who reported high numbers and growth rates of *Massilia* sp. in the presence of *Cryptomonas* sp. during co-cultivation in an artificial minimal medium.

Other members of *Cryptomonas*-associated cluster included Alphaproteobacteria, from the genus Tabrizicola *(Tabrizicola pisces*), and *Gemmobacter* sp. HYN0069 (Rhodobacteriaceae) is known to be associated with high-metabolic production of phytoplankton-derived organic matter [103,104]. Another member, the *Cryptomonas*-associated microbiome *Pseudobacter ginsenosidimutans* (Chitinophagaceae), has been reported to participate in plant decomposition [105].

### 3.3. Associated Microbiomes and Environmental Parameters

The composition of microbiome associated with *Microcystis* spp. has been previously linked to environmental factors such as temperature, seasonality, *Microcystis* morphology, and its density [55,106,107]. Additionally, this composition changes during seasonal bloom development and degradation. The effect of heatwaves was most studied on Cyanobacteria, with summer heatwaves typically promoting cyanobacterial blooms [108,109]. The microbiome bacteria associated with *Microcystis* exhibit a functional potential that is not found within the *Microcystis* itself [13]. Our results indicate that the microbiomes linked to different *Microcystis* morphospecies are quite resilient to environmental influences, which aligns with previous findings of Gobler and Jankowiak [110]. We identified temperature and shifts in nutrient concentrations (phosphate) as critical factors in microbial community composition, in particular, affecting microbiomes at the IPCC A2+50% regime but not at the AMB regime, where *Microcystis* prevailed.

The legitimacy of extending mesocosm findings to natural freshwater habitats and water bodies—sources of drinking water—to management decisions was discussed since opponents of mesocosm findings state that mesocosm experiment results do not account for long-term changes in ecosystem dynamics [111,112]. In our experimental mesocosm settings, we mimicked temporary heatwaves affecting thermal stratification and shaping algal blooms. Together with other researchers, we argue that experimental tests of the effects of mesocosm size on ecosystem responses produce important insight into drivers of complex ecological processes and are important for water management decisions [113,114]. Our findings, derived from climate change-relevant temperature regimes (IPCC A2 and IPCC A2+50%), offer valuable insights into how future warming and altered stratification may impact community dynamics in shallow lakes. Moreover, the combined use of full-length *16S rRNA* sequencing and IFC provides a framework for high-resolution monitoring, which could support early detection of bloom events and the development of targeted remediation strategies.

## 4. Conclusions

In freshwater bodies that serve as sources of drinking water for human use, it is crucial to understand the mechanisms that shape microbial communities and the health risks caused by the mass development of potentially toxic Cyanobacteria, and to explore the potential strategies for harmful algal bloom control.

A detailed species-level analysis of microbiomes, conducted using long-read nanopore sequencing alongside the quantitative dynamics of major phytoplankton groups, revealed a complex structure within associated microbial communities. As indicated by ordination-based analyses and co-occurrence-based topological inference, thermal stratified communities were significantly different in terms of their microbial and phytoplankton compositions. We identified temperature and shifts in nutrient concentrations, specifically phosphate, as critical factors influencing microbial community composition. This effect was particularly significant in the IPCC A2+50% regime, but not in the AMB regime, where *Microcystis* dominated. Alphaproteobacteria and Chitinophagia microbial classes were found to be more abundant during stratification periods, while an increased abundance of Ignavibacteria and Sphingobacteria was recorded during the mixing period. These findings suggest an additional factor influencing microbial community composition—temporary thermal stratification. Our results also highlight the importance of species-level analysis, as the varying compositions of *Pseudomonas*, *Hydrogenophaga*, and *Porphyrobacter* genera across treatments were recorded only at the species and not genus level. Furthermore, positively associated microbial clusters were identified and characterized in relation to each of the *Microcystis* morphospecies, including potentially toxic. These findings provide a foundation for future studies exploring climate-driven phytoplankton and microbial dynamics and support the development of high-resolution monitoring strategies. A combination of visualization tools (IFC) and long-sequencing approaches are important in evaluation of the dynamic microbiomes and their effects on planktonic behavior that is emerging to be a fundamental rule of life.

## 5. Materials and Methods

### 5.1. Collection of Mesocosm Samples

Water samples for analysis were collected at Aarhus University’s AQUACOSM Lake Mesocosm Warming Experiment (AU LMWE) facility in Silkeborg, Denmark [115]. To investigate how heatwaves affect the ecosystems of shallow lakes, the AU LMWE 2021 experimental setup simulated a heatwave-induced thermal stratification lasting for two weeks. The thermal stratification was achieved by altering the mixing patterns within the mesocosm tanks (i.e., switching off mixing paddles and lowering the level of heating elements). In total, 14 days of stratification were followed by 14 days of mixing during the summer period of July–August 2021. We followed the dynamics of microbial–microalgal communities in three high-nutrient tanks: D1—ambient (AMB), D2—IPCC A2, and D3—IPCC A2+50% climate scenarios. Water samples were collected for two months (once per week for eight weeks) with two sampling points—the surface and bottom of the tank. Weeks 1, 2, 5, and 6 correspond to stratification periods, and weeks 3, 4, 7, and 8 to mixing periods. A schematic overview of the experimental workflow is presented in Suppl. Figure S4.

### 5.2. Environmental DNA Extraction and Sequencing Library Preparation

Filtration of water samples for sequencing analysis was followed by DNA extraction. For this purpose, DNEasy Power Water Kit (Qiagen, Hilden, Germany) was used to extract DNA from filters [116] following procedures provided by the manufacturer (with an additional lysis step with heating. The full-length 16S gene was amplified using universal 16S primer pair: 27F (5′-AGAGTTTGATCCTGGCTCAG-3′) and 1492R (5′-TACGGYTACCTTGTTACGACTT-3′). Library preparation was conducted according to the methodology provided by the manufacturer (ONT, Oxford, UK). The PCR-reaction mixture consisted of nuclease-free water, input DNA, LongAmp Hot Start Taq 2X Master Mix (New England Biolaboratories, Ipswich, MA, USA), and respective 16S barcode primer. PCR products were then cleaned using AMPure XP beads (Beckman Coulter, Brea, CA, USA). The final step of library preparation included the addition of sequencing adapters to the mixture of barcoded samples. Following library preparation, the MinION Mk1C (ONT) was used for the sequencing run (40–46 h).

### 5.3. Bioinformatic Analysis

Raw signal data were stored in FAST5 files and underwent basecalling using Guppy (ver.6.5.7) neural network-based basecaller integrated into the MinION Mk1C device (ONT). The obtained FASTQ files were then processed using Python commands, starting with assessing the reads and their quality using the NanoPlot package (ver. 1.44.1) (https://github.com/wdecoster/NanoPlot) (accessed on 27 August 2024). This step was followed by quality and read length filtering, with a minimum quality score set of 10, followed by removal of adapter sequences and demultiplexing of reads into respective barcodes. The demultiplexed reads were then classified using the Emu taxonomic abundance estimator up to the species level (https://gitlab.com/treangenlab/emu) (accessed on 27 August 2024) [117,118]. Obtained data was rarefied before further statistical analysis.

### 5.4. Imaging Flow Cytometry-Based Analysis of Phytoplankton Community Composition

**Figure 12:**
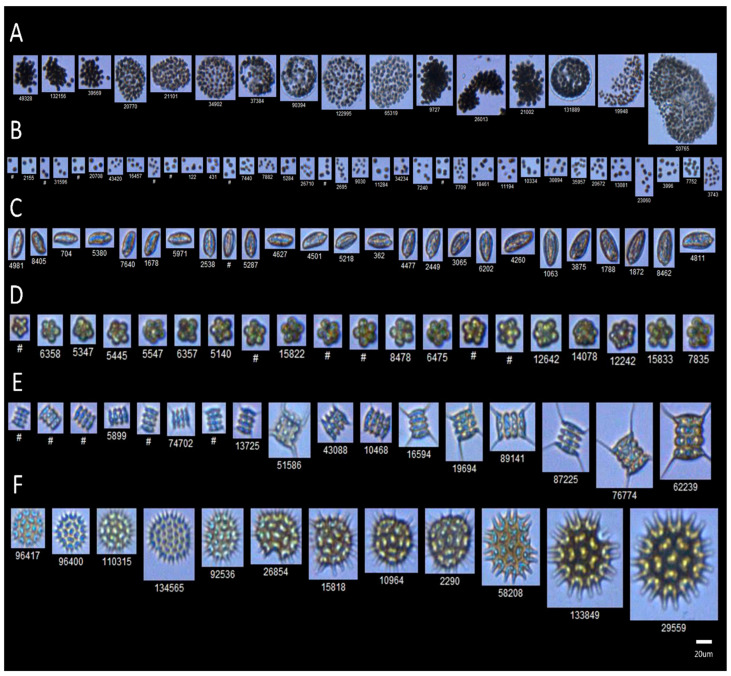
FlowCAM-based classification of major phytoplankton groups from the AU LMWE experiment. (**A**) Colonial *Microcystis* sp.; (**B**) non-colonial small clusters (NCSC); (**C**) *Cryptomonas* sp.; (**D**) *Micractinium* sp.; (**E**) *Scenedesmus* sp.; (**F**) *Pediastrum* sp.

The collected samples were analyzed in parallel with IFC using a benchtop FlowCAM VS-4 imaging flow cytometer (Yokogawa Fluid Imaging Technologies, Scarborough, ME, USA). Water samples fixed with 1% glutaraldehyde were analyzed with an autoimage mode using a 10X objective, followed by manual classification using VisualSpreadSheet (version 4.15.1) software. The major phytoplankton groups identified in this study are depicted in Figure 12.

### 5.5. Statistical Analysis

R packages vegan (vs. 2.6-4), ggplot2 (vs. 3.4.3), and GraphPad Prism software (vs. 9; Dotmatics, USA) were used for the analysis and visualization of obtained results. Diversity indices, specifically alpha-diversity metrics, were used to characterize microbial communities within and between samples, which include observed richness and Shannon index. In addition, beta diversity metrics (Bray–Curtis dissimilarity) were assessed to quantify dissimilarity between communities and were further subjected to non-metric multidimensional scaling (NMDS) [119,120,121]. A pairwise analysis of similarities (ANOSIM) was then applied to test the significance of the differences. Co-occurrence networks were constructed using Spearman and Pearson coefficient-based correlation matrices. Reads were first filtered to eliminate species with less than 0.5% relative abundance, and obtained correlation coefficients <0.70 were then filtered out (*p*-value < 0.05) prior to network visualization using the R package corrplot (version 0.92). Networks were then visualized and clustered using the R package igraph (vs. 1.5.1), and Gephi software vs.0.10.1 [122]. Fruchterman Reingold layout was used for visualization of all obtained networks. Node-level topology statistics were analyzed using R package ggpubr (vs.0.6.0). Canonical correspondence analysis (CCA) was used to describe the relationship between microbial community composition and environmental variables. In addition to this, indicator species analysis was conducted to define microbial species of interest associated with each treatment [120,121]; indicspecies (vs1.7.14) R-package was used for this purpose.

## Data Availability

Raw sequence data are deposited in the National Center for Biotechnology Information (NCBI) Sequence Read Archive (SRA) under the BioProject ID PRJNA1012663.

## Supplementary Materials

The following supporting information can be downloaded at: https://www.mdpi.com/article/10.3390/toxins17080370/s1, Figure S1: Environmental parameters throughout the experiment for the D tanks; Daily temperature levels (left panel) for surface (purple), middle (blue), and bottom (green) layers in tanks D1, D2, and D3; daily oxygen levels (right panel) for surface (blue) and bottom (red) layers in tanks D1, D2, and D3. Figure S2: FlowCAM-based classification of *Microcystis* morphospecies from the AU LMWE experiment; (A) *M. wesenbergii*; (B) *M. smithii*; (C) *M. ichtyoblabe*; (D) *M.novacekii*; (E) *M. aeruginosa*; (F) non-colonial small clusters. Figure S3: Relative abundance of top 12 microbial classes in tank D1 with AMB temperature regime across contrasting mixing/stratification periods; Figure S4: Schematic overview of experimental workflow; Sample collection, filtration and hydrochemical analysis were done at LMWE mesocosm location (University of Aarhus, Denmark). DNA extraction, library preparation, next generation sequencing and imaging flow cytometry analysis were performed at N.S.B. laboratory (Nazarbayev University, Kazakhstan); Table S1: Phylum composition of co-occurrence networks at varying temperature regimes; Table S2: The species composition of microbial clusters with significant correlations (*p*-value < 0.05, r > |0.7|) with *Microcystis* morphospecies in the surface layers of tank D1; Table S3: The species composition of microbial clusters with significant correlations (*p*-value < 0.05, r > 0.75) with Microcystis morphospecies in the bottom layers of tank D1.

## Abbreviations

The following abbreviations are used in this manuscript:

DNA	Deoxyribonucleic acid
Std Dev	Standard deviation
SRA	Sequence read archive
rRNA	Ribosomal ribonucleic acid
PCR	Polymerase chain reaction
OTU(s)	Operational taxonomic unit(s)
ONT	Oxford nanopore technologies
NMDS	Non-metric multidimensional scaling
NGS	Next-generation sequencing
NCSC	Non-colonial small clusters
mNGS-IFC	Microbial NGS combined with imaging cytometry
IPCC	Intergovernmental Panel on Climate Change
IFC	Imaging flow cytometry
HAB	Harmful algal bloom
FCM	Flow cytometry
CCA	Canonical correspondence analysis
ANOSIM	Analysis of Similarities
AMB	Ambient temperature regime
