# Supplementary material for: Heatwave-Induced Thermal Stratification Shaping Microbial-Algal Communities Under Different Climate Scenarios as Revealed by Long-Read Sequencing and Imaging Flow Cytometry
Source: Toxins (Basel). 2025 Jul 27;17(8):370. doi: 10.3390/toxins17080370 (PMC12389774; doi:10.3390/toxins17080370)
Supplement: Supplementary file 1 [file toxins-17-00370-s001.zip › toxins-3745533-supplementary.pdf]

Heatwave-Induced Thermal Stratification Shaping Microbial-Algal Communities Under Different Climate Scenarios as Revealed by Long-Read Sequencing and Imaging Flow Cytometry

Ayagoz Meirkhanova [1,*], Adina Zhumakhanova [1], Polina Len [1], Christian Schoenbach [1,†], Eti Ester Levi [2], Erik Jeppesen [2,3,4,5,6], Thomas A. Davidson[2] and Natasha S. Barteneva [1,7,*]

1 Department of Biology, School of Sciences and Humanities, Nazarbayev University, 010000 Astana, Kazakhstan; adina.zhumakhanova@nu.edu.kz (A.Z.); polina.len@alumni.nu.edu.kz (P.L.)
2 Department of Ecoscience, Aarhus University & Center for Water Technology (WATEC), 8000 Aarhus, Denmark; eel@ecos.au.dk (E.E.L.); ej@bios.au.dk (E.J.); thd@bios.au.dk (T.A.D.)
3 Sino-Danish Centre for Education and Research, Beijing 100049, China
4 Department of Biological Sciences and Centre for Ecosystem Research and Implementation, Middle East Technical University, 33731 Erdemli-Mersin, Turkey
5 Institute of Marine Sciences, Middle East Technical University, 33731 Erdemli-Mersin, Turkey
6 Institute for Ecological Research and Pollution Control of Plateau Lakes, School of Ecology and Environmental Science, Yunnan University, Kunming 650500, China
7 The Environmental Research and Efficiency Cluster, Nazarbayev University, 010000 Astana, Kazakhstan
* Correspondence: ayagoz.meirkhanova@nu.edu.kz (A.M.); natalie.barteneva@nu.edu.kz (N.S.B.)
† Deceased.

Received: 23 June 2025
Revised: 14 July 2025
Accepted: 25 July 2025
Published: 27 July 2025

Citation: Meirkhanova, A.; Zhumakhanova, A.; Len, P.; Schoenbach, C.; Levi, E.E.; Jeppesen, E.; Davidson, T.A.; Barteneva, N.S. Heatwave-Induced Thermal Stratification Shaping Microbial-Algal Communities Under Different Climate Scenarios as Revealed by Long-Read Sequencing and Imaging Flow Cytometry. *Toxins* **2025**, *17*, 370. https://doi.org/10.3390/toxins17080370

Copyright: © 2025 by the authors. Licensee MDPI, Basel, Switzerland. This article is an open access article distributed under the terms and conditions of the Creative Commons Attribution (CC BY) license (https://creativecommons.org/licenses/by/4.0/).

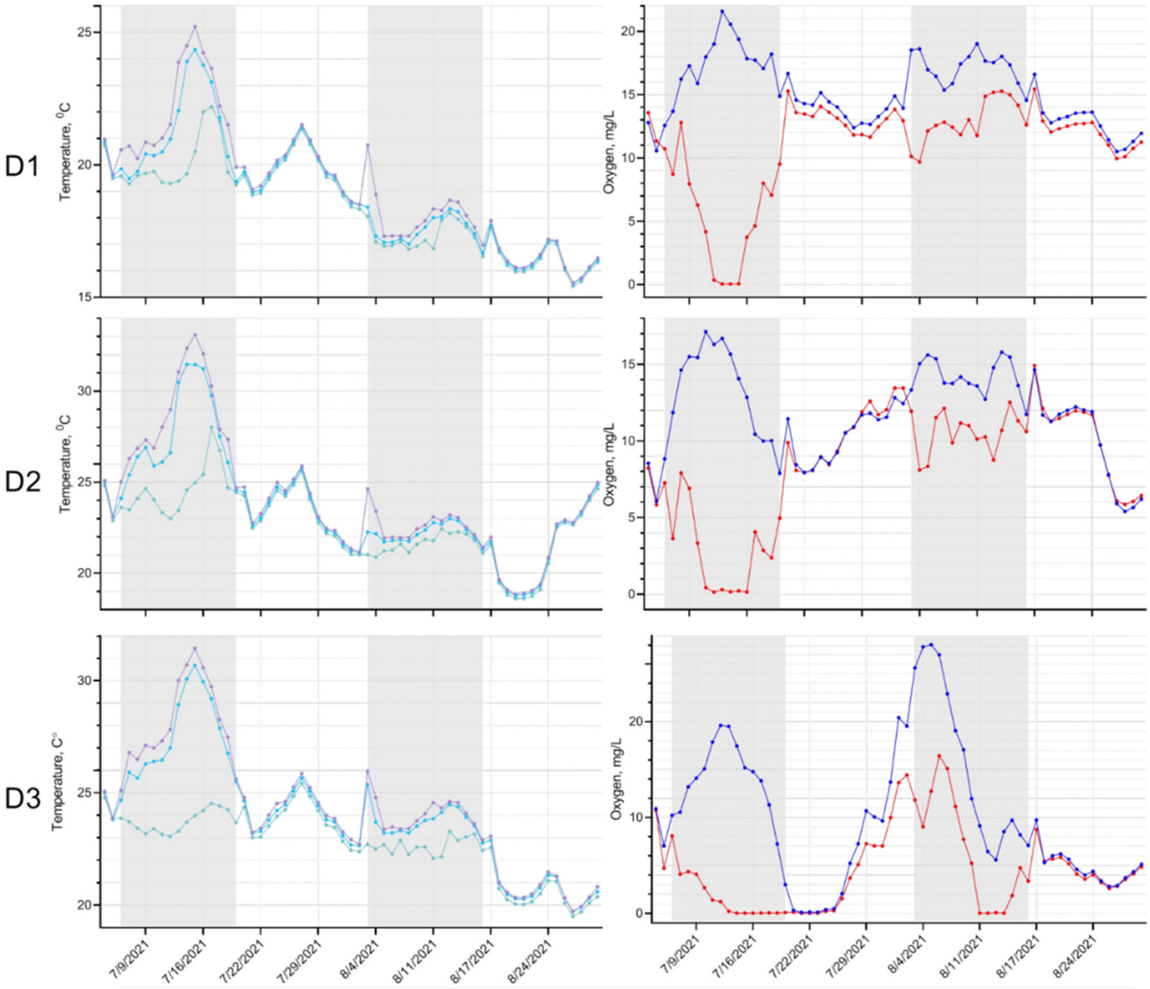

Supplementary Figure S1. Environmental parameters throughout the experiment for the D tanks. Daily temperature levels (left panel) for surface (purple), middle (blue), and bottom (green) layers in tanks D1, D2, and D3; daily oxygen levels (right panel) for surface (blue) and bottom (red) layers in tanks D1, D2, and D3.

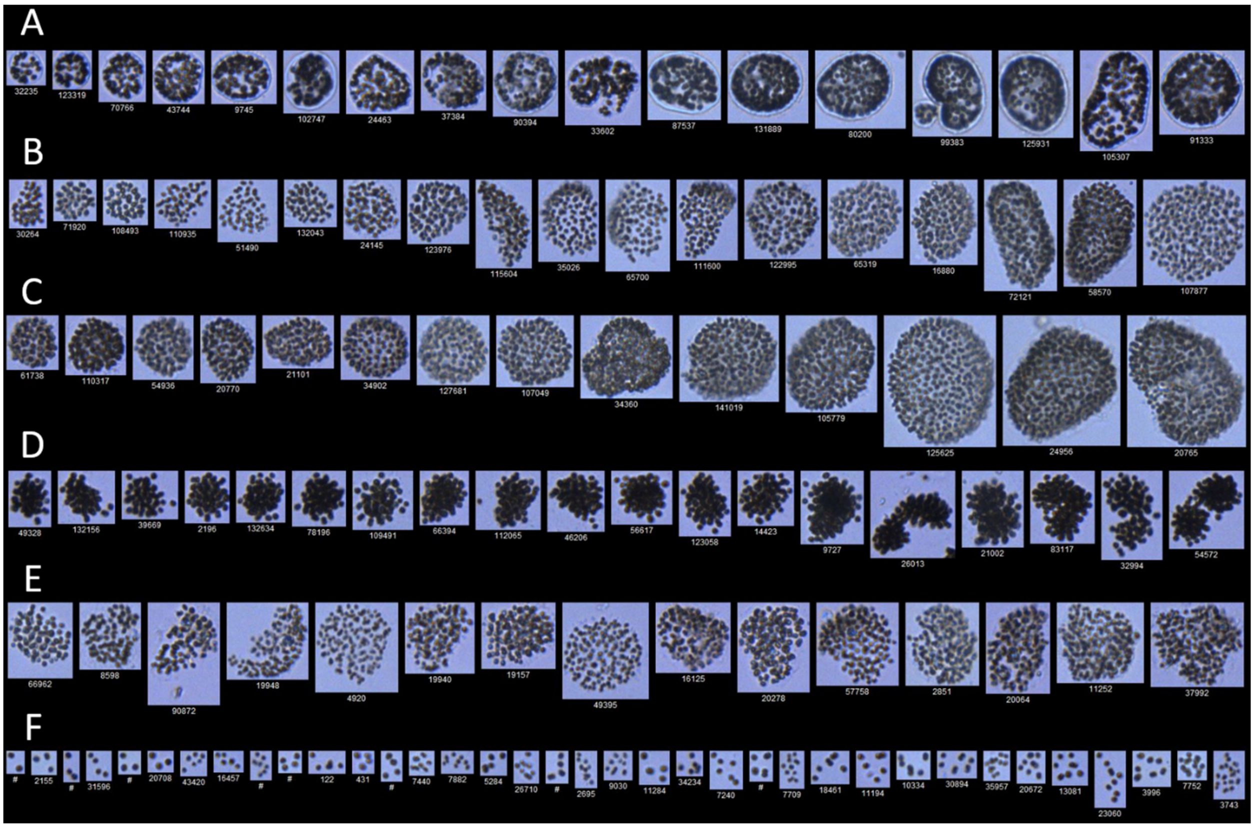

Supplementary Figure S2. FlowCAM-based classification of Microcystis morphospecies from the AU LMWE experiment. (A) *M. wesenbergii*; (B) *M. smithii*; (C) *M. ichtyoblabe*; (D) *M.novacekii*; (E) *M. aeruginosa*; (F) non-colonial small clusters

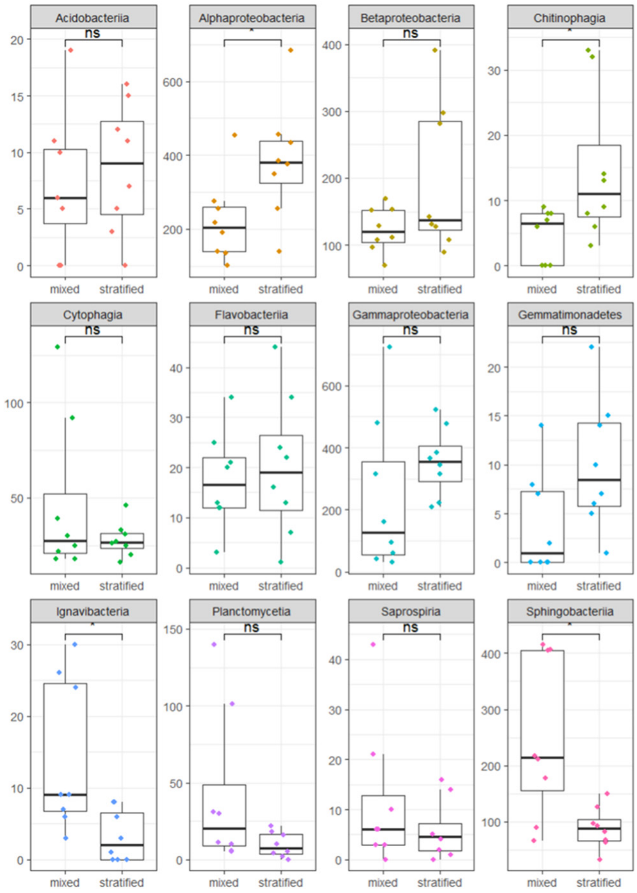

Supplementary Figure S3. Relative abundance of top 12 microbial classes in tank D1 with AMB temperature regime across contrasting mixing/stratification periods.

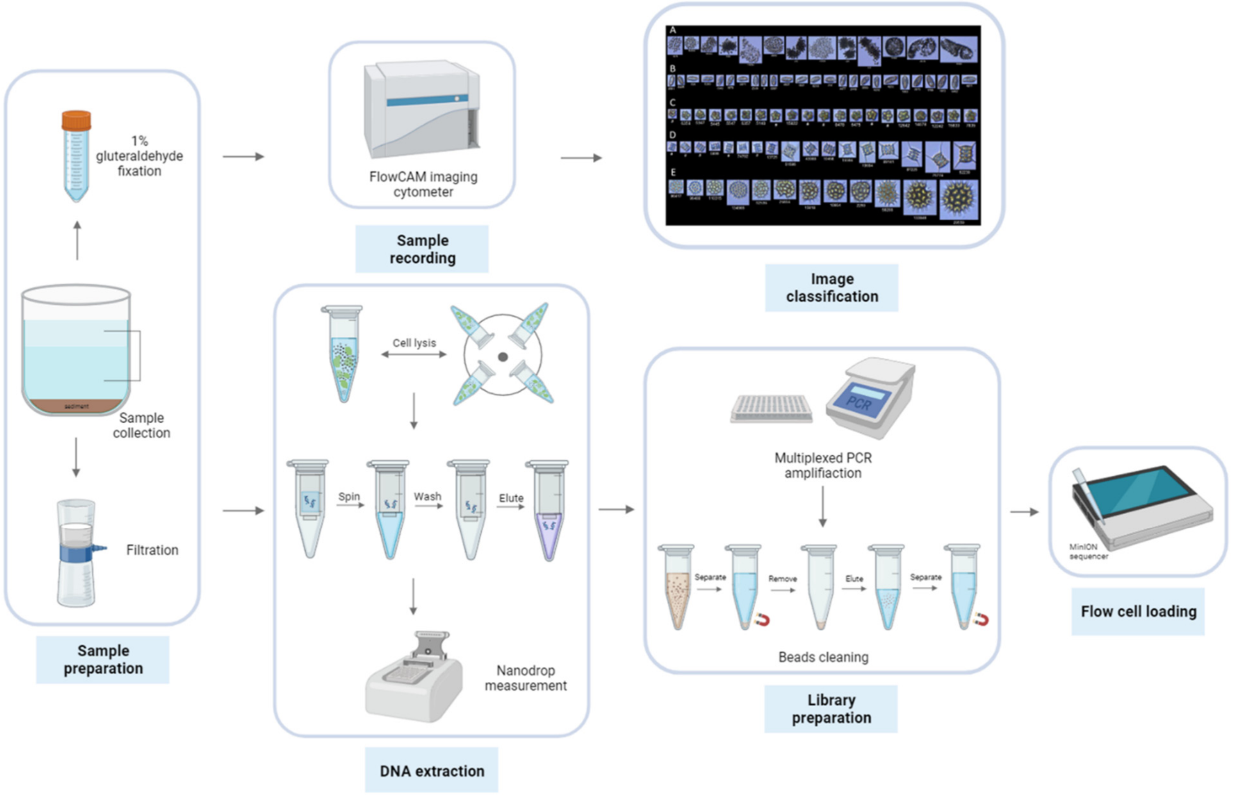

Supplementary Figure S4. Schematic overview of experimental workflow. Sample collection, filtration and hydrochemical analysis were done at LMWE mesocosm location (University of Aarhus, Denmark). DNA extraction, library preparation, next generation sequencing and imaging flow cytometry analysis were performed at N.S.B. laboratory (Nazarbayev University, Kazakhstan).

Supplementary Table S1. Phylum composition of co-occurrence networks at varying temperature regimes.

	AMB	IPCC A2	IPCC A2+50%
Proteobacteria	58.91%	66.91%	75.37%
Bacteroidetes	24.03%	20.14%	17.91%
Cyanobacteria	7.75%	6.47%	2.24%
Planctomycetes	3.88%	0.72%	
Gemmatimonadetes	1.55%	0.72%	
Acidobacteria	1.55%		
Firmicutes	0.78%	2.88%	0.75%
Verrucomicrobia	0.78%		1.49%
Ignavibacteriae	0.78%	0.72%	0.75%
Actinobacteria		1.44%	0.75%
Chlorobi			

Supplementary Table S2. The species composition of microbial clusters with significant correlations (p-value<0.05, r>|0.7|) with *Microcystis* morphospecies in the surface layers of tank D1.

Cluster	Class	Order	Species
3	Alphaproteobacteria	Caulobacterales	*Phenylobacterium muchangponense*
			Phenylobacterium sp. HYN0004
			Brevundimonas sp. M20
		Rhizobiales	*Methylocystis sp. SC2*
		Sphingomonadales	*Sphingopyxis sp. LPB0140*
	Betaproteobacteria	Burkholderiales	*Rubrivivax gelatinosus*
			Polynucleobacter difficilis
	Cytophagia	Cytophagales	*Cytophaga hutchinsonii*
			Chryseolinea soli
	Gammaproteobacteria		*Pseudohongiella spirulinae*
	Flavobacteriia	Flavobacteriales	*Flavobacterium buctense*
	Ignavibacteria	Ignavibacteriales	*Ignavibacterium album*
	Planctomycetia	Gemmatales	*Gemmata sp. SH-PL17*
		Isosphaeralis	*Aquisphaera giovannonii*
		Pirellulales	*Pirellula sp. SH-Sr6A*
		Planctomycetales	*Planctomyces sp. SH-PL62*
	Sphingobacteriia	Sphingobacteriales	*Solitalea canadensis*
			Pedobacter cryoconitis
			Sphingobacterium kitahiroshimense
			Solitalea koreensis
			Mucilaginibacter gossypii
			Daejeonella oryzae

			Mucilaginibacter sp. PAMC 26640<br>*Mucilaginibacter polytrichastri*<br>*Sphingobacterium sp. ML3W*<br>*Pedobacter sp. PACM 27299*<br>*Mucilaginibacter rubeus*<br>*Pedobacter mongoliensis*
		Nostocales	*Calothrix parietina*<br>*Dulcicalothrix necridiiformans*<br>*Iphinoe spelaeobios*
		Chroococcales	*Candidatus Atelocyanobacterium thalassa*
		Oscillatoriales	*Geitlerinema sp. PCC 7407*
		Chroococcidiopsidales	*Aliterella antarctica*
4	Acidobacteriia	Bryobacterales	*Bryobacter aggregatus*
	Alphaproteobacteria	Holosporales	*Candidatus Paracaedibacter acanthamoebae*
		Rhodobacterales	*Tabrizicola piscis*<br>*Tabrizicola aquatica*<br>*Pseudorhodobacter psychrotolerans*
		Rhodospirillales	*Roseomonas stagni*<br>*Roseomonas wooponensis*
		Sphingomonadales	*Novosphingobium subterraneum*<br>*Porphyrobacter sanguineus*<br>*Porphyrobacter colymbi*<br>*Blastomonas fulva*<br>*Sphingomonas piscinae*
	Betaproteobacteria	Burkholderiales	*Hydrogenophaga taeniospiralis*<br>*Thiobacter subterraneus*
	Flavobacteriia	Flavobacteriales	*Flavobacterium cheniae*
	Gammaproteobacteria	Xanthomonadales	*Stenotrophomonas rhizophila*

Supplementary Table S3. The species composition of microbial clusters with significant correlations (p-value<0.05, r>0.75) with *Microcystis* morphospecies in the bottom layers of tank D1.

Cluster	Class	Order	Species
**1**	Alphaproteobacteria	Caulobacterales	*Phenylobacterium sp. HYN0004*<br>*Brevundimonas sp. M20*<br>*Phenylobacterium muchangponense*
	Betaproteobacteria	Burkholderiales<br>Nitrosomonadales	*Polynucleobacter difficilis*<br>*Methyloversatilis discipulorum*
	Cytophagia	Cytophagales	*Cytophaga hutchinsonii*<br>*Chryseolinea soli*
	Gammaproteobacteria		*Acidibacter ferrireducens*
	Epsilonproteobacteria	Campylobacterales	*Sulfuricurvum kujiense*
	Ignavibacteria	Ignavibacteriales	*Ignavibacterium album*
	Planctomycetia	Gemmatales<br>Isosphaerales	*Gemmata massiliana*<br>*Aquisphaera giovannonii*
	Saprospiria	Saprospirales	*Haliscomenobacter hydrossis*<br>*Phaeodactylibacter luteus*
	Sphingobacteriia	Sphingobacteriales	*Anseongella ginsenosidimutans*
		Nostocales	*Loriellopsis cavernicola*<br>*Dulcicalothrix necridiiformans*
**3**	Betaproteobacteria	Burkholderiales	*Rubrivivax gelatinosus*

Flavobacteriia	Flavobacteriales	*Flavobacterium buctense*
Planctomycetia	Pirellulales	*Pirellula sp. SH-Sr6A*
Saprospiria	Saprospirales	*Portibacter lacus*
Sphingobacteriia	Sphingobacteriales	*Daejeonella oryzae*<br>*Arcticibacter svalbardensis*<br>*Mucilaginibacter sp. PAMC 26640*<br>*Mucilaginibacter polytrichastri*<br>*Sphingobacterium sp. ML3W*<br>*Mucilaginibacter gotjawali*<br>*Pedobacter sp. PACM 27299*<br>*Pedobacter mongoliensis*<br>*Solitalea canadensis*<br>*Pedobacter cryoconitis*<br>*Sphingobacterium kitahiroshimense*<br>*Mucilaginibacter gossypii*
	Nostocales	*Calothrix parietina*
	Chroococcales	*Candidatus Atelocyanobacterium thalassa*
